# Beyond legislation and technological design: The importance and implications of institutional trust for privacy issues of digital contact tracing

**DOI:** 10.3389/fdgth.2022.916809

**Published:** 2022-09-27

**Authors:** Yan Teng, Yan Song

**Affiliations:** ^1^ Ethics and Philosophy of Technology Section, Delft University of Technology, Delft, Netherlands; ^2^ Chair of Environmental Technology and Design, Delft University of Technology, Delft, Netherlands

**Keywords:** trust, privacy, digital contact tracing, digital health, digital ethics, COVID-19

## Abstract

For proper implementation of digital contact tracing technologies for fighting against SARS-CoV-2, participants' privacy vulnerability and the uncertainty from the relevant institutions' side could be seen as two core elements that should be dealt with, among others. In this paper, we propose to understand the current approaches for preserving privacy, referred to as privacy by legislation and privacy by technological design, as distrusting strategies that primarily work to reduce participants' vulnerability by specifying and implementing privacy standards related to this digital solution. We point out that mere distrusting strategies are insufficient for the ethically appropriate development of this digital solution, nor can they eliminate the need for institutional trust that plays an essential role in fostering voluntary support for this solution. To reach well-grounded trust in both an ethical and epistemological sense, we argue that trust in institutions concerning personal data protection in the case of digital contact tracing ought to be built on the relevant institutions' and individuals' goodwill towards the public and their competence in improving the actual effectiveness of this solution. We conclude by clarifying three dimensions, including the purpose, procedure, and outcome, where the relevant trustees can work to signal and justify their intentions and increase their trustworthiness *via* an effective communication strategy. Given the complementary qualities shown by the distrusting and trusting strategies, a combined strategy including both sorts seems closer to what we expect from the responsible implementation of this digital solution, which could also improve the effectiveness of this institutional response.

## Introduction: the deficit of institutional trust as part of the privacy issues

Contact tracing is a crucial way to break the chain of transmission (1, 2). It helps find and notify people who have been in close proximity to symptomatic and asymptomatic patients to take further measures (e.g., test and self-quarantine). During the COVID-19 epidemic, however, the large number of pre-symptomatic infections and the fast speed of SARS-CoV-2 transmission have posed grave difficulties in doing this manually (3). By using instant signals of smartphones, digital technologies promise to improve the efficacy of the tracing processes by minimizing the time to find, notify, and quarantine the contacts at risk (4). Much research has shown that digital contact tracing technologies, as a supplement to conventional tracing measures and part of a system of containment measures, can play a positive role in strategies for easing intense lockdown measures against the virus spreading (5–8). Wymant et al., for example, investigate the epidemiological influence of NHS COVID-19 on England and Wales (9).^1^ The result shows that in the three months since the app launched online, the app has averted 100,000 to 900,000 potential infections.

Along with the prominence achieved by the efficacy promise of digital contact tracing, privacy concerns have been voiced about potential misuse, information disclosure, and digital surveillance. After a wide-ranging debate about privacy risks, democratic societies—though to a limited extent—are in rough agreement on developing privacy-preserving digital contact tracing apps (10). However, the low uptake of many privacy-preserving initiatives shows an intractable issue of this digital solution: citizens' general lack of trust in institutions with respect to personal data protection (11, 12).

According to Ogury's research, more than half of the respondents in the US, France, and the UK state that they do not trust their government to protect any data they share through the tracing apps (13). The apps developed and used in these countries contain distinct settings, and their privacy risks remain controversial. Take the example of data storage methods. The majority of the apps applied in the US (e.g., WA Notify in Washington and CA COVID Notify in California) and the UK (e.g., ProtectScotland and NHS Covid-19) use a decentralized protocol that only stores anonymized data on users' phones, intending to preclude violations by design (14). In contrast, France's primary contact tracing app—TousAntiCovid—utilizes centralized servers to aggregate data. The government advocates that Google/Apple's widely-used decentralized system is not sufficient for protecting privacy because ephemeral identifiers of diagnosed users are accessible to other users and such sensitive data can be used to identify patients (15). Given these distinctions, Ogury's research concludes that users' trust in government needs to be rebuilt no matter which basic technique a tracing app is built on.

In fact, the developer community not merely includes governments. The public-private partnership that also involves technology companies and research institutions is common practice (16). It won't be surprising that people are more apprehensive about digital tracing when private, commercial companies are stepping in and functioning as a separate “data controller” from, or even a “gatekeeper” for public agencies. Considering their profit-minded shareholders and notorious records of data breaches, as Bradford et al. put forward, any uncertainty health providers and patients hold towards the future use of the medical data shared through or issued by third parties could discourage the uptake of the apps, making people constantly skeptical about the privacy promises made by these institutions on their tracing apps (17).

The fact that current privacy-preserving apps fall short of fostering residents' participation delivers a more profound public concern over institutions' intentions of promoting the apps. Namely, people are anxious that the relevant institutions and power holders are promoting the tracing technologies primarily for business and political interests instead of putting the citizens' best at heart. Such a worry can lead to a severe challenge to the tracing apps; namely, people may hardly trust that the institutions will protect their data even though many high privacy requirements and promises of data protection have been made publicly. As Kreps et al. point out, in the absence of institutional trust, citizens may *not* perceive the privacy-preserving apps as privacy-preserving and thus obviate the apps to control unwanted privacy losses (18). Combined with the significant role of trust in impacting the adoption of new technologies shown by various empirical studies (19–21), the low uptake of those tracing initiatives with a lower level of trust seems to be a coherent result.

Taking the above impacts of trust into consideration, we provide a novel perspective to examine privacy issues related to contact tracing technologies by viewing the lack of institutional trust as part of, rather than an additional issue that is separate from, users' privacy anxieties associated with this institutional response. A holistic understanding of distinct strategies and approaches that help address users' privacy concerns is provided, with a particular focus on clarifying the importance and implications of moral trust relations based on goodwill. As such, rather than asking general questions of trust, we start from the descriptive aspect of institutional trust (i.e., its impacts on technology adoption) and then delve into the discussion on its normative aspect (i.e., what makes justified trust decisions), seeking to explore what is at stake morally in the relation between citizens and institutions beyond the privacy issues on the surface, and the potential ways that can help release such tension.

## Exiting lockdowns: why or why not digital contact tracing?

While it is clear that, without specific therapeutics, any measure used for easing intense lockdowns (e.g., the closure of business and movement restrictions) should be assessed cautiously, it is unclear which set of measures is most effective. One formula that seems successful when used in the first phase of the COVID-19 outbreak in Singapore, China, and South Korea is a three-step approach called “test-trace-isolate” (22). After experiencing the first wave and intense lockdowns, these countries were grappling with the next step. Thus, the key purpose of this approach is to allow the gradual reopening of economic and social activities in the prevention of overwhelming the health care system and a potential next wave. Each of the three steps is considered crucial and indispensable. According to Aleta et al., for example, a resurgence of the epidemic can be prevented when 50% of symptomatic cases are identified by tests, 40% of their contacts are traced, and all of the contacts are then quarantined for two weeks (23). In a comparative study done by Panovska-Griffiths et al., these figures are 59%–87%, 40%, and 75% respectively (24).

### Digital contact tracing and its role in the overall strategy

Considering the intractable features of SARS-CoV-2, while the conventional way of tracing relying on tracers remains necessary in the fight, it is shown to be insufficient to find enough contacts without delays (4). This means that other supplement tools for tracing are needed in order to make the overall strategy useful (3). As mentioned, digital technologies based on smartphones might play a role here. Despite that the more citizens use the apps, the more effective the apps might perform, the efficacy of digital tracing apps is not a binary off-on switch. Research has shown that digital tracing combined with other containment measures can contribute to the reduction of infections, deaths, and hospitalizations at almost any level of uptake rate (8, 25, 26). For example, even only 15% of people use the apps, according to Abuge et al.'s model, they can reduce around 8% of infections and 6% of deaths (7). Thus, the goals of digital tracing are quite clear: (1) to find the contacts being overlooked by traditional tracing and contribute to making the overall three-step approach to be more useful; and (2) to provide rapid notification of exposures to reduce delays occurring between individuals being exposed and being tested or quarantined.

Considering the potential benefits of digital tracing, by April 2021, more than 90 countries have launched their mobile-assisted tracing apps (27). Most of these apps implement digital tracing by two technologies: Global Positioning System (GPS) and Bluetooth (low energy mode). Apps that use GPS technology collect users' location data and use a central server to analyze whether the location information of the app users overlays with the spots of those positively tested patients at a similar time (28). The apps will then alert the direct or indirect contacts accordingly. Apps that use Bluetooth seek to achieve a similar goal of alerting potentially infected people but by swapping anonymous codes with other app users when they are nearby at a certain distance (e.g., 3 meters) for a certain period (e.g., 15 min) (29). Based on the code switch history, users will get a notification when their contacts upload a positive diagnosis. In terms of the general goals of digital tracing discussed, both technologies can contribute to finding more exposed people and shortening the time of notifying and isolating these people and their contacts.^2^

It should be noted that policy goals related to COVID rules are changing, and so does the role of digital contact tracing. As the virus and the global situations change over time, especially after the emergence of the more devastating variant—Delta—and the more fast-spreading variant—Omicron, many countries have eased non-pharmaceutical interventions such as mask-wearing, restricted unnecessary traveling, and social distancing. As of March 2022, the UK has already scrapped most hard restrictions as the country plans to “live with the virus”.^3^ Singapore, a country where 92% of the population completed a full vaccination regimen but still experienced the highest peak in February 2022, also shifts to regard the virus as endemic.^4^ But the change in the overall strategy does not mean that digital contact tracing is not useful. In both countries, the app remains important in the test-trace-isolate project.^5^ In Singapore, nearly all the population has participated in the nation's digital contact tracing program and it remains significant in the overall strategy. Their app—TraceTogether—is now used not just for regular contact tracing but also as an important tool for preventing the spread among those who are not eligible to have vaccines, such as the elderly (30).

Therefore, it can be said that the role played by contact tracing apps at different stages of the fight is different, depending on the pandemic situations and the overall public health decisions. Instead of being used as part of a system for quitting lockdowns while preventing a resurgence, these apps now become a normalized tool to reduce the number of infections and slow down the transmission. Also, it should be noted that these apps may still face some practical issues, such as false alarms, civil compliance to voluntary self-quarantine, and reliance on high-quality mobile devices (31–33). For the interest of this paper, in the next subsection, we focus on discussing the privacy concerns associated with early tracing initiatives.

### Privacy concerns over early tracing apps

To discuss these issues in good order, here we use the clarification of the privacy concept provided by Warnier et al. as a simple framework to structure our discussion (34). While there are different conceptions of the privacy concept in philosophy, the three interconnected aspects of privacy they propose seem to nicely capture the most intractable issues faced by poorly designed apps.

Consider first *freedom from intrusion*. Although none of the apps are compulsory to be downloaded, some are strictly linked to other aspects of human life, such as travel and entering public spaces. For instance, a QR Code used as verifiable digital proof of travel history or vaccination is widely adopted as an electronic certificate for activity permits in China and many EU countries. Similarly, tourists to South Korea are required to install Self-Check to report their health conditions through the app for 14 days after arriving (35). By binding app installation with permission for social activities, both cases are in tension with privacy as an effort to strive for freedom from external constraints and render the apps de-facto mandatory (36).

Consider second *the control of personal data*. Having control over information concerns the restriction of information flow and whether it flows properly (37). Tracing initiatives, such as Singapore's TraceTogether and Norway's early Smittestopp, apply central servers to store and analyze the uploaded anonymous data, which enables the authorities to gain more insight into epidemic responses. Nevertheless, data aggregation not only contains the risk of being hacked and divulged but also threatens users' right to control over the flow of personal data and increases the risk of “mission creep” since the authorities might abuse their power and illegitimately use the contact tracing data for other purposes such as law enforcement (38).

Consider third *freedom from surveillance*. Data gathered by central servers might also be used for surveillance purposes, particularly considering those initiatives that collect vast location data and unnecessary personal information, such as gender, age, and profession (14, 39). The comprehensive information collection makes it possible for the authorities to produce big-data-driven policies to mitigate or suppress the contagion. However, a combination of the behavior-related information (e.g., locations and payment history) and identity information can be illegitimately used to not only track, watch, and follow a specific person's movement and travel history but also analyze implicit information linked to other characteristics and inner lives of the data subject (e.g., sexual orientation).

In times of public health crisis, while it is clear that measures that could contribute to “flattening the curve” are urgently required, it remains unclear how much privacy should be traded off in the name of community needs and to what extent governments' expansion of surveillance power can be justified (40). Such trade-offs are inextricably linked to the social-political contexts to which the apps are applied. Nevertheless, some obvious privacy flaws, such as the collection of unnecessary information and the analysis of behavior-related information, should be avoided by any tracing initiative for the sake of reducing unnecessary privacy costs. The pragmatic and epistemic weakness of citizens arguably creates an obligation of institutions to ameliorate the imbalanced situation and prevent from taking more advantage of the participants. In the next section, we begin by introducing two sorts of strategies related to trust that can help assuage the tension between citizens and institutions caused by the adoption of the apps. With this structure, we then take a closer look at the prevalent approaches for addressing privacy concerns, setting the stage for analyzing the value and implications of institutional trust.

## Distrusting strategies: current approaches for reducing vulnerability

Essentially, the privacy issues discussed above concern two main elements: users' vulnerability related to personal data and the uncertainty about how relevant institutions may manage users' data. Relations that involve these two elements are exactly the situations where trust becomes most relevant (41–43). As an attitude of the trustor (X), trust typically develops in situations where X has the need or interest to rely on a trustee (Y) with respect to the fulfilment of a particular entrusted thing (Z), but X cannot fully control or predict the behavior of Y (44). Here Z and other potential losses of X caused by Y's behavior can be seen as the vulnerability of X, and the essential reason for X's vulnerable position is that X is uncertain about Y's real trustworthiness. These two commonalities indicate that the case of digital contact tracing is a plausible situation where citizens' trust in institutions can be relevant and cause real effects on app adoption.

### Two sorts of strategies related to trust

As Heimer (45) clarifies, there are two sorts of strategies that are particularly useful for facilitating more reliable interactions under conditions of vulnerability and uncertainty. The first is *trusting strategies* that seek to find more information about Y's competence and intentions to decrease uncertainty about Y's trustworthiness. If the information at hand suggests that Y is competent and bears goodwill towards X, X will likely trust Y to protect rather than harm the thing X cares about. The conception of trust used here assumes the trustee's goodwill as a basic characteristic of trust relations, which essentially distinguishes trust from reliance by justifying feelings of betrayal and the expectation that Y will take X's vulnerability into account favorably (46, 47). Conversely, if finding enough information is not available or costs too much time, energy, and resources, people might opt for *distrusting strategies* that strive to limit others' untrustworthy actions and reduce the vulnerability of themselves, for example, by making contracts, more specific market access standards, and terms for sanctions and compensation. These measures, when serving the purpose of limiting improper actions, provide warranties and guarantees to participants who have a stake in the interaction, leading to compliance and reliance that are often used as alternative or complementary approaches to trustworthiness and trust (48).

Perhaps in stark contrast to the folk understanding of trust and distrust, scholars working on trust often interpret these two concepts as *functional equivalents* (43, 49). Essentially, both of them are ways of managing risks and benefits of social relations for the sake of reaching favorable results (50). Social structures, as Lewicki et al. argue, tend to be healthier and more stable when high levels of trust and distrust coexist (51). Thus, not merely do trust and distrust are considered not opposites, but also the tension between trust and distrust is understood as useful and productive for the relationship between citizens and institutions. In particular, distrusting strategies and proper regulations can in fact help create more trust in a relatively safe environment.

In the context of digital tracing, getting sufficient information about the relevant institutions and individuals' trustworthiness seems not easy for ordinary people. This is because many citizens lack the knowledge and capability to rationally assess the relevant entities' competence, nor can they easily find ways to be aware of the actual intentions of these entities. In most cases, ordinary people cannot even find someone to whom their uncertainty can be directed due to the complex division of labor in such a nation-state-based or transnational solution. This also explains why in modern societies, strict measures, like legislation, contracts, and insurance, that do not rely on one's familiarities with another's intentions and competence are used more often among strangers (52).

We argue that the prevalent approaches adopted to address the privacy issues, referred roughly to as privacy by legislation and privacy by technology (as we will discuss below), are closer to distrusting strategies rather than trusting strategies. The essential idea of these two approaches is to utilize legal and technical means to specify and implement a complex set of privacy requirements, such as data parsimony and data anonymization, formalizing the way that users' vulnerability can be reduced in the context of digital contact tracing. Here users' vulnerability is the direct and indirect information-related risk engendered by using the apps, including harms, injustice, and inequalities caused by the disclosure of diagnosis information or other data issued by the apps. As the question of what personal data might be at stake is largely determined by the kind of underlying technologies chosen by different apps and a complex set of criteria applied to regulate the life cycle of the apps, these two approaches can be crucial ways to ameliorate users' vulnerability.

### Privacy by legislation and privacy by technological design

Privacy by legislation refers to the idea of protecting participants' vulnerability by the enactment, enforcement, and optimization of data protection laws and regulations. While poorly designed digital tracing apps pose serious threats to users' personal data, stringent privacy laws and regulations make app developers, data controllers, data issuers, and other relevant entities to be legally bound to create privacy-preserving apps to avoid lawsuits, fines, fees, and the loss of reputation (53, 54).

In the EU context, for example, digital tracing falls into the General Data Protection Regulation's (GDPR) comprehensive scope that requires system design of digital tracing to demonstrate: lawfulness, fairness, and transparency; purpose limitation; data minimization; accuracy; storage limitation; integrity and confidentiality; and accountability [(55) Art.5]. The regulation's expansive scope and principle-based approach, as Bradford et al. argue, offer a ready-made and flexible functional guideline for creating new technology applications that protect basic human rights (17). The Pan-European Privacy-Preserving Proximity Tracing is a fundamental effort to translate GDPR's general rules into more detailed technical standards for guiding the design and development of tracing apps in the EU context (56).

To some extent, privacy by technological design can be seen as a means of implementing privacy laws, but it is more than that since design can also be used to incorporate various norms and values into the product (57). Can we make the design of the tracing apps more ethically appropriate beyond what is required by laws and regulations? Based on the previous introduction of the core underlying technologies that enable a tracing app, it can be said that Bluetooth plus local data storage embroil fewer privacy costs than other options since the former set collects little identifiable data. Table 1 provides a review of different technical settings concerning the apps mentioned throughout this paper. Based on their digital tracing technology and contact history storing, the apps can be categorized into four types (see Table 1).

**Table 1:** A comparison of different apps on their basic technology.

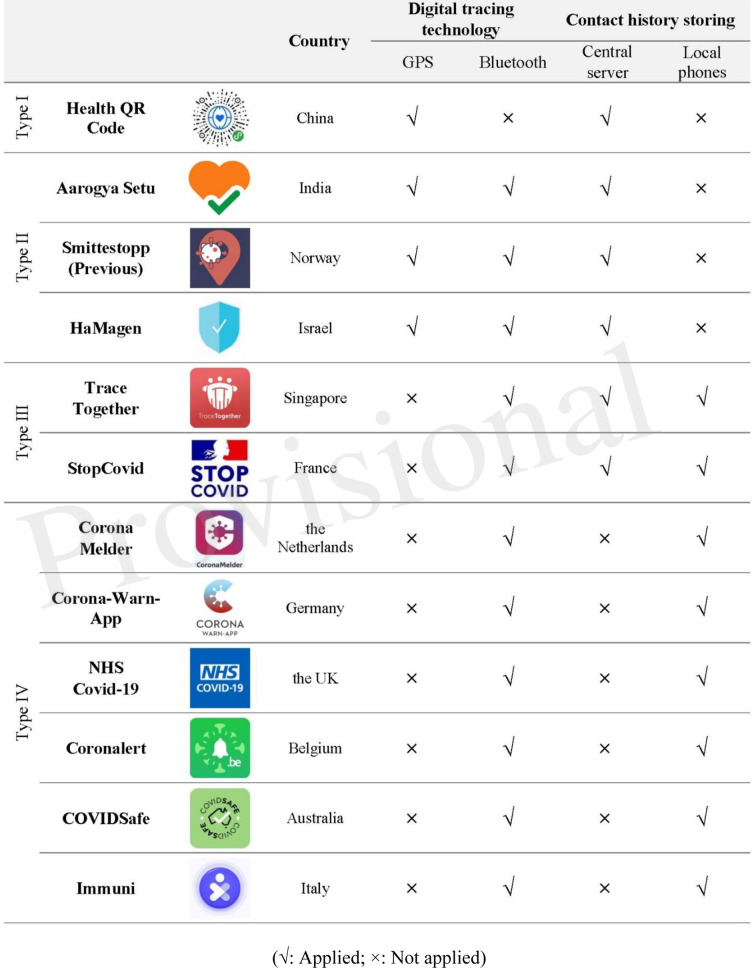

To be more specific, while the appropriate use of location data collected by GPS-based apps relies heavily on legal constraints, industrial standards, and central authorities' responsibility for processing data in a lawful and secure manner, Bluetooth-based apps weaken the identifiability at the technology level without the aid of legislation and bureaucratic structures. For this reason, Bluetooth-based apps could be considered a product of both privacy by legislation and technological design. Likewise, while data protection in centralized servers relies heavily on privacy laws and regulations, decentralized databases that keep the exchanged identifiers merely on users' phones can reduce the reliance on centralized organizations and bureaucratic structures to protect data, which could also be regarded as a product of both privacy by legislation and privacy by technology.

Granted, many privacy concerns over the violation of users' freedom from intrusion, control of information, and freedom from surveillance present by early tracing initiatives have been addressed by opt-in, Bluetooth-, and decentralization-based contact tracing apps together with other institutional privacy assurances. The uptake rate of such a privacy-preserving solution seems not as high as expected, even though it has already been higher than that of more intrusive solutions.^6^ Many citizens' privacy anxieties still exist, despite the apps' vulnerability-reducing settings and the recommendation of participation appealed by public health authorities, governments, and privacy experts.

## Dealing with uncertainties: institutional trust and trustworthy institutions

While the current approaches discussed are necessary for providing a good starting point for proper implementation of digital contact tracing, they are not in themselves sufficient to facilitate the uptake of contact tracing technologies, nor can they eliminate the role of trust in fostering or impeding widespread voluntary adoption of digital contact tracing technologies. Besides, there is a danger that the situation of trust deficit may be exacerbated by the distrusting strategies adopted. As O'Neill (52) and Thompson (58) point out, trying to increase uptake merely by using regulatory approaches to limit some untrustworthy conduct shows the very idea of “economizing on trust”, which may squeeze out the role played by trust, including its positive correlation with technology adoption.

An important reason for the insufficiency of current approaches is that although many privacy standards have been set and put into practice, there is a wide variety of nuanced, implicit, and unforeseen situations that may engender privacy risks but have yet to be covered by regulatory measures and technological solutions. For example, once diagnosis information is divulged, a broad sense of social avoidance, discrimination, bias, and other information-based harms might be imposed on the infectious, and some of these harms can neither be fully addressed nor equally compensated by the above measures. This means, even though the measures discussed can mitigate the power imbalance between citizens and power holders by specifying and regulating the latter's actions, being participants still directly points to people's privacy-related vulnerabilities that they would otherwise not take.

Following Heimer's (45) and Kerasidou (48)'s identification discussed, the uncertainties of the institutions' side involved in the case of digital contact tracing are just the places where warranted institutional trust can play a role in encouraging uptake. That is to say, other things being equal, people will likely *only* choose to participate and cooperate with those institutions that favorably take into account their vulnerabilities and act as counted on; namely, those institutions that they think are trustworthy and can really trust.

Understanding the value of trust provides an essential step toward the establishment of healthy citizen-to-institution relations. Citizens' trust is a good thing for institutions to implement pandemic responses, but it should be clear that trust is not something that can be enforced or demanded. The only way to gain or regain trust is by improving the potential trustee's trustworthiness, which makes trust easier to flourish (59). From the trustor's perspective, although citizens generally have the need for being protected at the collective level (60), being too trusting comes with a considerable risk of generating false expectations and losing the entrusted things. As Devine et al. state, people may naively believe that institutions are doing the right thing or doing things in the right way when they are not (61). Due to the moral sensitivity of the entrusted things (e.g., illness history) and the irreversible harm that might be inflicted on data subjects once trust is frustrated, trust in the case of digital contact tracing should not be seen as something that can be unreflectively developed. Instead, a critical, ethical view of trust should be supposed.

Considering the above bilateral need for trust in the pandemic context, the normative question of what makes an institution trustworthy is of importance for both parties. To answer this question, we need to explicate what elements well-grounded trust ought to concern in the case of digital tracing and how such opinions can be applied to the improvement of institutions' trustworthiness, with the practical goals of making trust more warranted and the outcome of contact tracing technologies more morally desirable.

The first element, which is also the core one, is the associated trustees' *motives* for privacy protection in the case of digital contact tracing. In moral philosophy, trust is often considered as a distinctive concept assuming that the trustee bears goodwill towards the trustor and would like to take the trustor's vulnerability and dependence as compelling reasons for acting responsively, whereas reliance does not require so and is seen as a mere rational-decision based on the result of risk-benefit assessment (46, 62, 63). In the case of digital tracing, a distinction can thus be made between preserving privacy as an instrument for achieving other ends set by the relevant institutions and preserving privacy out of genuine care that sees individuals' privacy rights as part of the desired end. Viewing privacy as something intrinsically valuable and worthy of promoting and preserving fundamentally explains why by trusting, people (X) feel optimistic that the associated institution and individual representatives (Y) are committed to protecting their personal data (Z) issued by the app related to Y even in situations out of the protection of current legal and technical solutions, since they believe that the trustees will take their vulnerabilities into account favorably and act as counted on.

From this perspective, creating law-compliance tracing apps out of some morally controversial reasons, such as self-interest, fear of sanctions or opprobrium, and force of social constraints, seems not sufficient to guarantee the institutions' future trustworthy conduct. Reliable actions with motivation open to different contexts might be enough for citizens to interact with institutions in regular situations; however, in the pandemic context where people are already worried and anxious about their surroundings, more benign motives are arguably needed to improve the predictability of the outcome and comfort the sense of insecurity caused by the turbulence. Furthermore, motives governed by business practices and market thinking, when applied to other social spheres (e.g., public health), may jeopardize or simply crowd out non-material social good and moral values internal to those particular spheres (40, 57), resulting in a violation of justice and equality that further washes away the desirable grounds for building trust. Likewise, politicians and government leaders are criticized for putting political interests ahead of what the public cares about (64, 65). As Floridi (66) points out, in some cases, the development of the apps is not motivated from a public health standpoint. Still, it is rather a mere political solution that signals to the public that power holders have tried everything they can and should not be blamed for not trying.

These commercial and political opportunisms can raise the public's fear that the privacy promises and the actions that appear to be trustworthy are just means for achieving other ends of those power holders, which might be broken at a certain point. In fact, scandals of data breaches have been witnessed several times in the case of digital proximity tracings, such as North Dakota's tracing app where studies find that personal data has been sent to Google and other service providers with the app's privacy promises being ignored (67). Similarly, Israel's national security agency is reported to have the power to access the database of Israel's tracing app HaMagen for surveillance purposes despite the app's promise that users' data will not be transmitted to third parties (68). Such promise-breaking incidents may further undermine the public's image of the tracing apps in general. The moral apprehension about what motivates one to make a privacy promise is real. Such a concern, combined with the gradually strict rules adopted to regulate untrustworthy actions, may create a circular, self-reinforcing atmosphere of distrust that leaves little space for trust to thrive.

The second element constitutive of well-grounded trust concerns the awareness of the relevant trustees' *competence*. The evaluation of such competence mainly includes two aspects: whether users' personal data is well protected by a privacy-preserving app and whether the app is effective in achieving the predefined functional goals. While it is difficult for normal users to detect privacy problems until experts find loopholes or the spread of data breach news, the latter ultimately concerns whether developers and policymakers can sufficiently justify the effectiveness of, and the societal need for, contact tracing apps. It is important to note that the discussion about the function and role of digital tracing technologies provided in early sections is more about the app's efficacy—i.e., how well an app works in a controlled environment, instead of its effectiveness that considers how well the same app will work when it is released in a real-world situation. As Floridi (66) points out, the privacy issues and effectiveness of the apps, together with other ethical difficulties, need to be carefully assessed by a clear deadline so that we could determine how this digital project ought to be improved, renewed, or terminated. Meanwhile, the relational and situational nature of trust indicates that very often the goods of trust are not inextricably linked to a particular trustee or a particular means used by that trustee (69). For this reason, proper justification of the need for the apps should also include a comparison result between a contact tracing app with other alternatives contingent on different contexts and new opportunities.

Based on the will-centered account of institutional trust discussed above, for participants to trust an institution and the associated app, it means that they believe that the institution (1) does care about users' health and privacy right and develop the digital project as a means to improve citizens' well-being; and (2) would like to take possible steps to justify the need for, and improve the effectiveness of, the contact tracing app. Understanding institutional trust in this way does not lead to the fact that this trust is fully warranted given that trust is never fully warranted. Rather, this interpretation sketches the main value and meaning of trust as a complementary approach to legal frameworks and technological solutions. It captures the general expectation we have about what is appropriate for others to do and our shared sense of insecurity about others' motivation that is multiplied by the public health crisis.

## Implications of trusting strategies for digital contact tracing

Till now, we have discussed two sorts of strategies that can be used to reduce the privacy issues related to digital contact tracing and help facilitate interactions between citizens and institutions. To enhance readability, a framework of how these strategies are applied to this digital project is provided in Table 2. While it is clear that the choice of strategies largely depends on the context to which they will be applied and probably no countries purely use one kind of the strategies, trusting strategies emphasizing institutions and their individual representatives' intentions have received much less attention than the other type. In this section, we discuss how our moral opinions about institutional trust can be applied to the case of digital contact tracing to restore trust gradually through an improvement of institutions' trustworthiness. Combined with the distrusting approaches articulated, a combined strategy based on all the useful embodiments related to the two strategies seems closer to what we expect from the responsible implementation of this digital solution.

**Table 2:** A framework for the two sorts of strategies in the case of digital contact tracing.

	Central idea	Embodiments	Institutions	The public
Distrusting strategies	Reducing users’ vulnerability	Privacy by legislation	Comply with privacy policies, laws, and regulations	Be aware of one’s legal rights
		Privacy by technological design	Reduce privacy risks through technological innovations	Be aware of privacy implications made by different technologies
Trusting strategies	Reducing uncertainty about institutions’ trustworthiness	Intentions	Display genuine care towards public health and privacy rights	Get information about the potential trustee’s intentions
		Competence	Justify the effectiveness of and societal need for the apps	Get information about the potential trustee's competence

We propose three dimensions where institutions and their individual representatives can apply the will-centered trust account to increase their trustworthiness, highlighting the importance of an effective communication strategy that can be applied throughout the development and implementation processes of the apps. The first is the purpose dimension. The public's moral apprehension about what drives institutions to foster this digital project urges government and corporate leaders, employees, and app developers and maintainers to be willing and able reliably to show their intentions. To show care towards participants and the society at large, the relevant entities need to answer (1) why the development, deployment, and use of contact tracing apps can be considered a collective effort that can bring positive impacts on pandemic mitigation, and (2) how the apps could improve the well-being of individual participants without improper intrusion into their right to be left alone. Answering these questions justifiably would require the relevant entities to provide reliable and understandable information to the public, and demonstrate how their benign intentions will be used to inform the operation processes explicitly. A structured, consistent, and evidence-based communication strategy could help achieve the above goals. This considers, as Rehse and Tremöhlenb illustrate (70), reliable communicators, communication channels, information content, the timing of communication, etc. For the developers and supporters of the apps, open communication with the public requires the key actors to explicate the limited purposes, benefits, and temporary nature of the apps, including but not limited to an explanation of the necessity and proportionality of using digital contact tracing and an explanation of the relevant entities' restrictions and responsibilities such as data minimization, security, and retention (71, 72).

The second is the procedure dimension. Procedural values—such as transparency, fairness, solidarity, reciprocity, and accountability—that are often linked to good institutional responses are considered valuable for developing trust in the context of digital contact tracing (36, 73). Arguably, what makes the will-centered account of trust distinctive is its emphasis on the show of willingness to negotiate, compromise, and cooperate during the decision-making process. Public trust is not generated in an environment where the public's voice is not heard, even though that environment contains well-established legal frameworks and institutional procedures. Mechanisms built on the willingness to negotiate directly facilitate communication by shifting a certain level of control from power holders to those who are less powerful, enabling the latter to relieve some burden and anxieties of the former's discretionary power over the actual result of trust. Some governments that secure trust successfully during the epidemic have already shown the usefulness of such trusting strategies. For example, public agencies in Taiwan have built multiple platforms that allow citizens to participate in the enactment of public policies, such as the distribution of medical supplies (74). The inclusive and interactive ideas involved not just deliver that the authorities do care about citizens' interests and would like to implement the policy responses in a responsive manner, but they also inspire civic-mindedness and engagement that are considered crucial for fighting against the pandemic. Degeling et al. record six deliberative workshops held to investigate how people feel about the appropriateness of adopting digital contact training in Australia (75). After a series of rigorous discussions with experts and each other, the majority of all six groups support the current privacy protection standards adopted by COVIDSafe. For the public, open communication is an interactive way of not merely getting more information but also providing forthright feedback to the app processors. This allows different groups of people to cultivate a positive feedback loop where institutional trust can thrive.

The third is the outcome dimension. Probably the most straightforward way to justify the trustee's goodwill towards the trustor is to make the entrusted thing or task warranted, to honor rather than break the privacy promises that invited trust, to show honesty, empathy, and accountability by taking real actions and deliberating with the public, to improve the welfare of participants instead of making troubles by sending false alerts and misinformation. That also explains why the adoption rate of digital contact tracing technologies is likely to be higher in communities that have high trust in institutions before the pandemic (76). The outcome of trust can thus be seen as a vital evaluation standard of trust, which directly impacts whether one would like to continue or stop trusting. Nevertheless, this seems to indicate the difficulty of initiating a trust relation, which somehow comes back to the usefulness of distrusting strategies in facilitating tentative interactions by providing a relatively safe route for individuals to depend upon others while gathering information about others' real trustworthiness.

That is to say, in terms of how to deal with public health recommendations and governmental policy responses made for achieving collective goals, it might be helpful for citizens to start tentatively from distrusting strategies. For example, one may start by understanding the privacy implications of distinct technological settings used for contact tracing purposes, and by being aware of whether a given initiative defers to data-protection laws and industry self-regulation in advance. Meanwhile, institutions and their representatives should continue to reduce participants' vulnerability as well as signal and justify their intentions and competence, seeking to augment trustworthiness and decrease participants' uncertainties about the overall interaction. Later on, if that participant has sufficient successful experience with the interacted institution that also gains a fine reputation from the society, their distrust might turn into a trust that can lead to more effective group functioning and productive social activities.

## Conclusion

While trust, together with institutional procedures, technical settings, and market techniques, form the bedrock of cooperation in modern society, the absence of trust could create considerable difficulties in the execution of any public policy. During the pandemic, we have witnessed a fracturing of trust in many institutions worldwide, but a gradual recognition of the value of trust and the urgency of restoring trust. In this article, we have critically engaged with the topic of trust in institutions within the framework of the two sorts of strategies discussed. Distrusting strategies and trusting strategies, considering their central ideas, embodiments, and detailed implications for institutions and citizens, are not merely complementary to each other, but also both considered indispensable for the proper and effective implementation of contact tracing technologies. Despite that there are no easy ways to fix trust in a short time, institutions should understand how trust works and work to explicitly improve their trustworthiness.

## Footnotes

^1^NHS COVID-19 app was launched on 24 September 2020. By the end of 2020, this app was downloaded by 34.3 million people and approximately half of them use the app regularly (around 28% of the population).

^2^It should be noted that the apps may contain other purposes that hinge on the concrete design and functionality of the apps.

^3^For the COVID-19 response of the UK, see https://www.gov.uk/government/publications/covid-19-response-living-with-covid-19/covid-19-response-living-with-covid-19.

^4^To see the vaccination rate in Singapore, please refer to https://www.statista.com/statistics/1223524/singapore-covid-19-vaccines-administered/.

^5^To see how the app is used in the UK, please refer to the website of Welsh government: https://gov.wales/nhs-covid-19-app#section-51119.

^6^For uptake of contact tracing initiatives in 2020, see https://craiedl.ca/gpaw/.
